# Pediatric Nasopharyngeal Cancer: Case Report and Review of the Literature

**DOI:** 10.7759/cureus.497

**Published:** 2016-02-15

**Authors:** Alejandro González-Motta, Garvin González, Yurany Bermudéz, Maria C Maldonado, Javier M Castañeda, David Lopéz, Martha Cotes-Mestre

**Affiliations:** 1 Department of Radiation Oncology, Instituto Nacional de Cancerologia, Universidad Militar Nueva Granada, Bogota, D.C., Colombia; 2 Department of Radiation Oncology, Instituto Nacional de Cancerologia, Universidad Militar Nueva Granada, Bogota, D.C., Colombia; 3 Clinical Research Group, Instituto Nacional de Cancerologia, Bogota, D.C., Colombia

**Keywords:** nasopharyngeal cancer, brachytherapy boost, chemoradiotherapy, pediatric nasopharyngeal carcinoma

## Abstract

Pediatric nasopharyngeal carcinoma, also referred to as cavum carcinoma, is a rare pediatric disease with an infrequent incidence rate. We present the case of a pediatric patient with nasopharyngeal cancer who received an adult schedule of concomitant chemotherapy and conformal radiotherapy with a brachytherapy boost. Adult protocols with high radiotherapy doses are not commonly used in pediatric patients due to the high comorbidity associated with this practice. In this case, the patient displayed excellent overall survival, a longer disease-free period, and fewer side effects and comorbidities, even in the absence of interferon therapy, which is not easily available in low-income countries. In addition, this case shows that conformal radiotherapy and brachytherapy are options that can be used to escalate the radiotherapy dose and decrease side effects.

A 12-year-old female pediatric patient presented to our outpatient clinic with an eight-month history of moderate-to-severe otalgia, intermittent hyaline rhinorrhea, asthenia, adynamia, nasal congestion, epistaxis in the previous months, and local pruritus. Upon physical examination, a 60x60 mm mass was detected at level II of the neck, and a biopsy of the lesion confirmed a histopathological diagnosis of undifferentiated carcinoma compatible with nasopharyngeal carcinoma. The patient was considered to have clinical Stage III cancer, and she received an adult Al-Sarraf protocol with chemoradiotherapy and an intracavitary brachytherapy boost. The patient had a complete response, and she remains without local or distance relapse.

Treating pediatric nasopharyngeal carcinoma patients with the Al-Sarraf protocol could be a feasible modality, as observed in this clinical case, despite the elevated cost of using interferon-beta in low-income countries when using more advanced radiotherapy techniques such as conformal radiotherapy and now, modulated intensity radiotherapy. It should be noted that brachytherapy boosts should be used with caution in pediatric patients; the potential side effects should be weighed against improved local control.

## Introduction

Nasopharyngeal carcinoma, one of the few epithelial-origin tumors observed in children, is distinguished from the adult form of the disease by its association with Epstein–Barr virus (EBV) infection, undifferentiated histology, and high incidence of advanced locoregional compromise [1]. It rarely appears in children under 14 years of age, and the annual incidence rate in the United Kingdom is 0.25 cases per one million inhabitants [2]. Although enormous differences exist among races and geographical groups, nasopharyngeal carcinoma makes up 1–5% of all pediatric cancers and 20–50% of all primary malignant nasopharyngeal tumors in children [1, 3-5]. This disease has been linked to etiological factors such as infectious mononucleosis, consumption of food rich in nitrosamines, and genetic and epigenetic factors that have not yet been clarified. Herein, we examine the case of a pediatric patient with a confirmed histopathological diagnosis via biopsy of undifferentiated nasopharyngeal cancer clinical Stage III, who benefitted in terms of overall survival, disease-free survival, and side effects from treatment with concomitant chemoradiotherapy.

## Case presentation

A 12-year-old female pediatric patient, previously healthy and without past medical history, was examined at the National Cancer Institute of Colombia, Bogota, in August 2007. The patient, who was accompanied by her mother, was a resident of Rovira, Tolima, Colombia. She presented to our outpatient clinic with an eight-month history of moderate-to-severe otalgia, intermittent hyaline rhinorrhea, asthenia, adynamia, nasal congestion, nasal voice, epistaxis in previous months, and local pruritus. Upon physical examination, a 60x60 mm mass was detected at level II of the neck with moderate, deep-layer adherence and signs suggestive of cervical adenomegaly. The patient complained of secondary mass-related pain, which had received adequate medical attention. A computerized tomography (CT) scan of the neck revealed a lesion that occupied the left cavum space, accompanied by cervical adenomegalies ipsilateral to the lesion. A cervical mass biopsy revealed undifferentiated carcinoma, and immunohistochemistry showed positivity for HLA DR, CK19, CK5/6, and EMA, without reactivity for CK7 and CK20 compatible nasopharyngeal carcinoma. Tests to detect EBV were requested, but no information was provided on the results. The patient was classified as clinical Stage III (T2N2M0) within the context of undifferentiated nasopharyngeal-type carcinoma.

**Figure 1:**
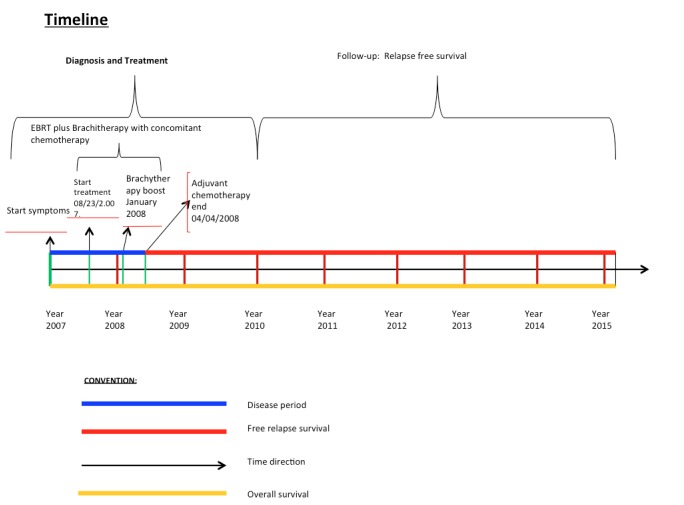
Timeline A year and a half after diagnosis and treatment, the patient has been without local or systemic relapse.

The patient was treated with three-dimensional conformal radiotherapy (3DCRT) with a fractionation of 2 Gy per day at planning target volume (PTV)42, consisting of the nasopharynx plus bilateral cervical drainage of regions Ib-II-III-IV-VII to 42 Gy with a boost up to 46 Gy at PTV46, consisting of bilateral cervical drainage of regions Ib-II-III-VII and nasopharynx. A resimulation CT scan was performed to provide a subsequent boost up to 70 Gy at PTV70 (macroscopic nasopharyngeal tumor plus left and right macroscopic cervical lymph nodes plus margin) concomitant with 100 mg/m^2 ^cisplatin on days 1, 21, and 42. Upon completion of the external radiotherapy, the patient had persisted with residual tumor in the nasopharynx. After discussing potential risks and associated morbidity, the patient was treated with a high-dose-rate brachytherapy boost to nasopharynx in two fractions, each 6 Gy at 1 cm from the applicator, then went to three subsequent adjuvant chemotherapy cycles with a single dose of 80 mg/m^2^ cisplatin on the first day of the cycle plus 1000 mg/m^2 ^ per day of 5-fluorouracil during days 1–4. Each cycle lasted 28 days (see Figure 1 for timeline).

**Figure 2:**
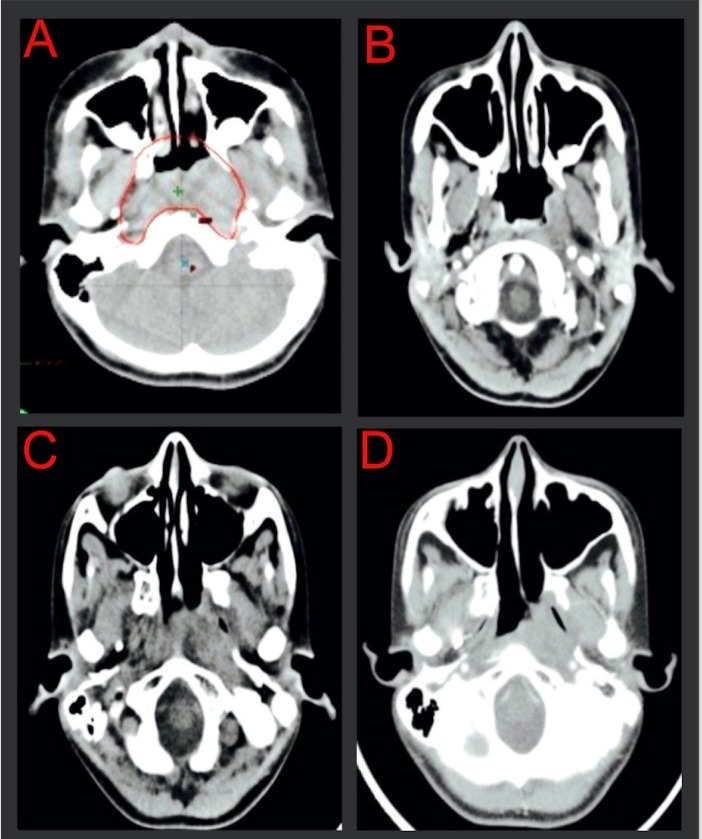
Simulation CT scan previous to treatment and control CT scans after treatment A) Simulation CT scan with delimitation of tumor in red. B) CT scan after finishing radiochemotherapy in 2007. C) Control CT scan in 2008 with abnormality in left nasopharyngeal fold due to post-radiotherapy fibrosis. D) Control CT scan in 2013 with persistent fibrosis without signs of malignant disease relapse.

**Figure 3:**
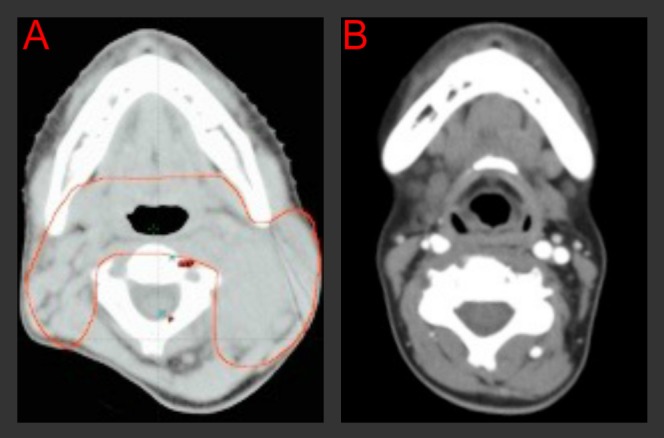
Tumor response from radiotherapy A. Simulation CT scan in 2007 shows in red level II lymphadenopathy. B. Control CT scan  in 2013 without local relapse.

The patient had a complete response to treatment without relapse (Figures 2, 3). However, after eight years of follow-up with an excellent local control, side effects have included chronic sinusitis, trismus Grade 2 and xerostomia Grade 1, multiple losses of teeth, nasal voice, and hypothyroidism. A CT scan of the neck performed in the first semester of 2015 revealed nasopharyngeal asymmetry; thickening of the left retropharyngeal space with irregular borders (observed on CT scan since one year after treatment completion, compatible with post-radiotherapy fibrosis without changes; Figure 3), with no consolidation or mass, no necrosis areas, and no bone erosion; and left choanal atresia obliteration. At the time of this writing, the patient is at university and works part-time.

## Discussion

Undifferentiated pediatric nasopharyngeal carcinoma is a common endemic tumor in southern China, Southeast Asia, the Mediterranean, and Alaska, but it rarely occurs in Japan, Europe, or North America [1]. In some areas of North America and the Mediterranean region, age distribution is bimodal, with an early incidence peak at 10–20 years of age and a second peak at 40–60 years of age [1]. Children under 16 years of age make up 1–2% of nasopharyngeal carcinoma cases in China, 10% in the United States, 12% in Israel, 13% in Kenya, 14.5% in Tunisia, and 18% in Uganda [1-2]. In the United Kingdom, the annual nasopharyngeal carcinoma incidence rate is 0.8 per million inhabitants 10–14 years of age, and 1–2 per million inhabitants 15–19 years of age [2]. The median age of nasopharyngeal occurrence in children is 13 years, with greater incidence in black males [1,6]. Genetic and environmental factors contribute to the development of nasopharyngeal carcinoma; it is linked to specific human leukemic antigens, chromosomal deletions, gene p53 inactivation, rearrangement of the retinoblastoma tumor suppression gene, and cadherin gene alterations [1,7-9]. EBV has been linked to the development of nasopharyngeal carcinomas, having been found in the tumor cell genome and in preinvasive dysplastic cells, thus suggesting that EBV is an early event in the carcinogenic process that produces the transformation of the epithelia and gives rise to nasopharyngeal carcinoma [1-2,10]. The pathogenesis of nasopharyngeal carcinoma in the pediatric population is considered to be a multiple-step process that includes EBV infection, which subsequently produces a simultaneous increase in the protein of the viral membrane that acts as a pro-oncogenic substance and in the p53 gene in epithelial cells [1, 11]. In accordance with the World Health Organization classification [12], the disease is grouped into three types: Type 1, keratinizing squamous cell carcinoma; Type II, non-keratinizing non-squamous cell carcinoma; and Type III, undifferentiated carcinoma. The latter is the most common pediatric variant [2]. Types II and III are linked to EBV, whereas Type I is not [13]. 

Nasopharyngeal carcinoma usually originates in the nasopharyngeal lateral wall, which includes the Rosenmuller fossa and extends into or outside of the nasopharynx, up to the contralateral wall or in a posterosuperior direction up to the cranial base or palate, nasal cavity, or oropharynx [2]. Metastasis frequently occurs in cervical lymph nodes [1-2, 8], due to abundant lymphatic drainage through the internal jugular chain, accessory spinal chain, and retropharyngeal space [1]. Distant metastasis usually appears in bones, lungs, mediastinum, and, much less frequently, in the liver [2]. In many patients, the initial appearance of nasopharyngeal carcinoma is a cervical adenopathy, and diagnosis is made with a lymph node biopsy [2]. Primary tumor symptomatology is similar to that of an influenza-like viral respiratory infection and can also present with trismus, painful mass, otitis media, nasal congestion, hearing loss, and cranial nerve paralysis secondary to skull base involvement [2]. Large tumor volumes produce nasal obstruction, bleeding, and nasal voice [2]. Differential diagnoses include upper respiratory tract diseases, non-Hodgkin’s lymphoma, Hodgkin’s disease, nasopharyngeal rhabdomyosarcoma, juvenile angiofibroma, germinal rhabdomyoma, and hemangioma tumors [1]. Diagnosis is further carried out through a careful clinical exam and imagological evaluation using CT scan with contrast to determine bone involvement and erosion, as well as magnetic resonance imaging to determine local tumor extension and cranial base compromise [1]. In addition, thorax X-rays and bone scintigraphy are taken in order to rule out distant compromise. The staging classification for nasopharyngeal carcinoma is based on the classification system of the Seventh Edition of the Joint American Committee on Cancer [14]. More than 80% of children with nasopharyngeal carcinoma are diagnosed at an advanced stage [6-8].

Optimum treatment in children has yet to be established; however, therapeutic strategies have been adapted from established treatment guides for adults, and the core treatment for pediatric, non-metastatic nasopharyngeal carcinoma is radiotherapy [15]. Due to anatomical localization and the tendency to exhibit cervical adenomegalies, pediatric patients are not appropriate surgery candidates for local control [2]. 

Several factors are taken into account when considering treatment with chemotherapy. Relapse-free survival is similar in the majority of chemotherapy series, but these series also include high-dose radiotherapy treatment, from 60 to 65 Gy, in the nasopharynx. Nevertheless, the most promising results have been achieved with the Mertens NPC-91-GPOH (German Society for Pediatric Oncological and Hematological) protocol [16], whose primary features include immunotherapy with interferon-beta (IFN-ß) following chemotherapy and radiotherapy, which might explain its superior results compared to regimens without interferon treatment [2, 17]. The Pediatric Oncology Group (POG) 9486 study used radiotherapy only for patients with T1-T2N0M0; and neoadjuvant chemotherapy with cisplatin, 5-fluorouracil, methotrexate, and leucovorin followed by radiotherapy for patients with T3-T4, and/or N1-N3, and/or M1 disease[17]. Possible late side effects of chemotherapy in children should also be taken into account; for example, the Manchester regimen consists of doxorubicin, methotrexate, and cyclophosphamide, and the side effects could be male infertility due to cyclophosphamide, as well as possible anthracycline toxicity [2]. The NPC-91-GPOH protocol might produce male infertility linked to cisplatin dosage; other potential side effects include renal toxicity and hearing loss [16].

The more recent NPC-2003-GPOH protocol [18] proposes only radiotherapy followed by adjuvant intravenous IFN-ß three times per week for six months for low-risk patients in Stages I and II. For high-risk patients, cisplatin plus folic acid plus adjuvant 5-fluorouracil is recommended for three cycles every 21 days, followed by radiotherapy and IFN-ß; during radiotherapy, cisplatin is administered three times during two cycles [18].

Undifferentiated nasopharyngeal carcinoma is highly sensitive to external radiotherapy treatment, thus making it the major pillar of the treatment [1]. Exclusive use of radiotherapy has led to survival rates of 20–60% at five years in several pediatric series [1, 6, 19-22]. The relationship between radiation dose and tumor response in children with nasopharyngeal carcinoma is debatable and difficult to establish due to the use of combined chemotherapy treatment [1]. Doses above 65 Gy have shown greater local control in a few studies [1]. The recommended dose varies between 50 and 72 Gy for primary tumors in patients over 10 years of age; the dose is reduced by 5–10% for children under 10 years of age [1, 5]. Conformational intensity-modulated radiation therapy has become increasingly accepted as the technique of choice in treating nasopharyngeal carcinoma in the adult population, as it allows for excellent coverage of target volumes, such as primary tumor, cranial base, and lymph node regions, with adequate protection of normal tissue adjacent to the treatment target including glands and other normal tissues [23]. In the pediatric population, it improves local control with fewer side effects, as demonstrated recently by Guo et al. [15], with overall survival, locoregional relapse-free survival, and progression-free survival rates of 90.8%, 94.9%, and 79.1%, respectively, at four years. It also results in lower incidence rates of xerostomia, hearing loss, and fibrosis compared with previous conventional radiotherapy studies. Due to the anatomical complexity of nasopharyngeal carcinomas, special care should be taken when planning treatment in order to avoid infradosification of the tumor margin zones, which could lead to tumor relapse [1]. In some patients, a 5–9 Gy boost to the primary tumor is administered with brachytherapy to achieve better local control [1].

In the German NPC-2003-GPOG protocol [18], the clinical volume target includes the primary tumor region and all visible macroscopic metastatic lymph nodes with a 1 cm margin, the entire nasopharynx, retropharyngeal lymph nodes, and cervical II level. In patients at disease Stages III and IV, the clinical volume target also includes lymph nodes at levels III, IV, and V, as well as the supraclavicular regions. In the German study, patients at Stages I and II received radiotherapy doses of up to 45 Gy in fractions of 1.8 Gy to the tumor at the nasopharynx and cervical lymph node drainage, followed by a 14.4 Gy boost to the tumor at the nasopharynx. Stage III and IV patients received radiotherapy at the nasopharynx and respective lymph node drainage, including the entire jugular group and supraclavicular region, with a total dose of up to 45 Gy in fractions of 1.8 Gy. The boost was 14.4 Gy, which was reduced to 9 Gy in patients with complete remission after neoadjuvant chemotherapy. Concomitant cisplatin was used over three consecutive days in the first and last weeks of radiotherapy.

A recent protocol from The Children's Oncology Group ARAR0331 uses radiotherapy alone for stages I and IIa to 61.2 Gy and 66.6 Gy, respectively [24]. For advanced disease uses neoadjuvant chemotherapy cisplatin and 5-flouracil followed by radiotherapy accord to response 61.2 Gy versus 70.2 Gy for responders and non-responders, respectively [24].

In terms of prognosis, 20–50% of patients experience relapse or metastatic disease, usually within 1–2 years after diagnosis; while a small proportion of patients with local relapse may be rescued, most metastatic patients die from the disease [1]. However, the prognosis has improved, thanks to advances in radiotherapy and the use of neoadjuvant or adjuvant chemotherapy, resulting in an event-free survival rate of 92.4% and overall survival rate of 97.1% with a median follow-up of 30 months in the NPC-2003-GPOH [18], and a four-year event-free survival and overall survival of 77% and 75% respectively in the POG 9486 [17]. In addition, the Guo et al., study reported a progression-free survival rate of 79.1% with the use of intensity-modulated radiation therapy [15].

As a rule, nasopharyngeal carcinoma treatment in adults involves radiotherapy and concomitant chemotherapy based on the Al-Sarraf schedule [25]. This schedule uses fractionated radiotherapy in doses of 2 Gy per day, up to a total dose of 70 Gy, with 100 mg/m^2 ^of concomitant cisplatin on days 1, 22, and 43 during radiotherapy; 80 mg/m^2^ of cisplatin following radiotherapy on days 1 and 5; and 1000 mg/m^2 ^per day of 5-fluoracil on days 1–4 for three cycles every four weeks. With this schedule, progression-free survival at three years has been calculated at 69% with a survival rate of 76%. However, this schedule is not commonly used in children due to radiation morbidity associated with high radiation doses. In children and adolescents, the neoadjuvant chemotherapy and subsequent radiotherapy schedule are preferred, but the best results are obtained with IFN-ß, which is expensive and is not widely available in low-income countries. There is currently no consensus regarding what the standard nasopharyngeal carcinoma treatment should be in children. In our case, the decision to deliver an adult schedule with high-dose radiation concomitant with chemotherapy to our pediatric patient was made due to a poor prognosis associated with nasopharyngeal carcinoma and the lack of availability of interferon at that moment. We also gave her a brachytherapy boost, and she achieved a complete local response, as shown in Figure 2.

## Conclusions

In the case described herein, the treatment chosen was the Al-Sarraf protocol with concomitant chemotherapy and radiotherapy frequently used to treat nasopharyngeal carcinoma in adults. It was considered to be a feasible modality in an adolescent patient, given the elevated cost of using IFN-ß. Concomitant chemoradiotherapy could be an excellent modality for the management of pediatric nasopharyngeal carcinoma, as observed in this clinical case, when 3DCRT radiotherapy or a more advanced technique is used to limit the dose to healthy tissues and organs at risk. However, brachytherapy boosts should be used with caution in pediatric patients; the potential side effects should be weighed against the possibility of improving local control. 
